# Congenital Heart Disease and Its Association in Children With Down Syndrome

**DOI:** 10.7759/cureus.29176

**Published:** 2022-09-14

**Authors:** Yasir Rehman, Haseen Dil Wazir, Ali Akbar, Abdul Moeed Khan, Ijaz Hussain, Amir Afridi, Huma Gul, Haleema Sadia

**Affiliations:** 1 Pediatric Cardiology, Peshawar Institute of Cardiology, Peshawar, PAK; 2 Pediatric Cardiology, Lady Reading Hospital MTI, Peshawar, PAK; 3 Medicine, Khyber Teaching Hospital, Peshawar, PAK

**Keywords:** congenital heart disease, ventricular septal defect (vsd), children, association, down's syndrome

## Abstract

Objective

The objective is to determine the frequency of different congenital heart diseases and their association in children with Down syndrome (DS).

Methodology

This cross-sectional observational study was conducted in the pediatric department of Peshawar Institute of Cardiology, Peshawar, Pakistan from August 2021 to July 2022. A total of 123 children with DS and congenital heart disease (CHD) were included in this cross-sectional study. Detailed history and examination were performed, and findings were documented on performed pro forma. Diagnosis of CHD was confirmed through two-dimensional (2D) and Doppler echocardiography performed by a pediatric cardiologist. Patients were managed according to standard protocols and guidelines. The data including age, gender, mother's age at the time of birth, type of CHD and growth failure were documented and analyzed. Percentages were used to express frequencies.

Results

The mean age was 2.2 years ± 3.4 years (interquartile range (IQR): 10 days to 14 years). There were 65 (52.8%) male and 58 (47.1%) female patients. Out of 123 patients, 101 (82.1%) had acyanotic CHD and 22 (17.8%) had cyanotic CHD. Among acyanotic CHD, isolated ventricular septal defect (VSD) was the most common observation in 23 (22.3%) and among cyanotic CHD, tetralogy of Fallot (TOF) in seven (31.8%) patients. The most common associations of CHD were VSD+ patent ductus arteriosus (PDA) in 11 (9%) patients and atrial septal defect (ASD)+PDA in nine (7.3%) patients. The median age of the mother at delivery was 31 years (interquartile range (IQR): 20 years to 45 years). Growth failure was seen in 70 (56.9%) patients.

Conclusion

Based on our data, VSD is the most common CHD followed by a complete atrio-VSD (CAVSD) in children with DS. The most common association of CHD in DS is VSD with PDA. Growth failure is seen in most of the patients with DS having a CHD.

## Introduction

Down syndrome (DS) is the most common chromosomal disorder in humans. It affects around one in 400-1,500 babies born in different populations depending on several factors [1]. Worldwide DS is a common genetic disorder causing intellectual disabilities and other systemic manifestations including congenital heart diseases (CHDs) [2]. In children with DS, CHD is the leading cause of mortality and morbidity under 2 years of age [3]. Almost 50% of newborn babies with DS have CHDs at birth [4]. DS patients undergoing cardiac surgery represent nearly 10% of total cardiac surgeries [5]. DS is associated with growth failure and growth failure is more significant if it is associated with CHD [6,7].

Previous studies have suggested the existence of ethnic variations in the type of CHD in the DS population [8]. It has been suggested by the studies that the profile of CHD in DS is different according to variable geographic areas of the world [9,10]. To improve preventive measures and provide quality health care, it is important to know the incidence of different types of CHD in DS and their associations. In addition, the type of CHD determines the timing of surgery and medical management in these children, which is important for optimal outcomes [11].

Limited data is published from South Asia in this regard. This study is aimed to find out the frequency of types of CHD, associated cardiac anomalies, and growth failure in children with DS at our hospital which is the only dedicated cardiac hospital in the Khyber Pakhtunkhwa province of Pakistan. This study will support efforts to enhance the screening of CHD in the pediatric population when DS is suspected and guide regarding preventive measures to avoid complications.

## Materials and methods

Study design

This descriptive cross-sectional study was conducted at the Peshawar Institute of Cardiology, Peshawar, Pakistan. Data collection was started in August 2021 and completed in July 2022.

Inclusion and exclusion criteria

All patients with down syndrome having congenital heart disease below 16 years of age were included. Hemodynamically insignificant lesions like patent foramen ovale (PFO) and tiny PDA less than one millimeter were excluded from the study.

Data collection

All the cases of DS with CHD who presented to the Department of Pediatric Cardiology were included in the study. All patients with a clinical diagnosis of DS (with or without chromosomal study) having CHD were included in the study. Confirmation of CHD was made on 2D and color doppler echocardiography performed by a pediatric cardiologist. Detailed history and examination were performed. CHD was classified as cyanotic and acyanotic CHD. Patients with a left to right shunting lesions were evaluated for pulmonary hypertension on echocardiography. Additionally, associated cardiac anomalies were also documented. Gender, age and nutritional status of the patient and maternal age at the time of birth were also documented.

Statistical analysis 

All numerical values obtained from each item of the data collection sheet as well as the demographic data were computed and presented by simple descriptive statistical tests, frequency, and percentage.

Ethical consideration

This study was approved by the hospital ethics committee, Peshawar institute of cardiology, Peshawar, Pakistan with the approval number HOD/06/127.

## Results

The total number of patients enrolled in the study was 123. Out of which, 65 (52.8%) patients were male, and 58 (47.2%) patients were female. The age range of cases was from 10 days to 14 years. The mean age was 26.7 months ± 41 months. The majority of the patients were diagnosed below one year of age (Table 1). Acyanotic CHD was the most common type of CHD found in 101 (82.1%) patients, while cyanotic CHD was found in 22 (17.8%) patients. The single cardiac lesion was seen in 67 (54.4%) patients while 56 (45.5%) patients had multiple cardiac lesions. Among patients having a single type of CHD, VSD was the most common type of CHD found in 23 (18.7%) patients followed by PDA and CAVSD in 18 (14.6%) and 11 (8.9%) patients, respectively. Isolated TOF was the most common type of cyanotic CHD found in seven (5.7%) patients. The most common associations of CHD were VSD+PDA in 11 (9%) patients and ASD+PDA in nine (7.3%) patients (Table 2). Severe pulmonary hypertension was seen in 56 (45.5%) patients. The median age of the mother at delivery was 31 years (20-45) (Table 3). Growth failure was seen in 70 (57%) patients. All patients with growth failure had acyanotic (left to right shunt) heart disease (Table 4).

**Table 1:** Age-wise distribution

Age	Number	Percentage
0 to 1 year	70	56.9%
1 to 5 years	40	32.5%
5 to 10 years	13	10.5%

**Table 2:** Types of congenital heart diseases Ventricular septal defect (VSD), Patent ductus arteriosus (PDA), Atrial septal defect (ASD), Pulmonary stenosis (PS), Complete atrioventricular septal defect (CAVSD), Double outlet right ventricle (DORV), Pulmonary atresia (PA).

Type of CHD	Number	Percentage
VSD	23	18.6%
VSD with associated PDA	11	8.9%
VSD with associated ASD and PDA	9	7.3%
VSD with associated ASD	6	4.8%
VSD with associated PSS	2	1.6%
PDA	18	14.6%
CAVSD	11	8.9%
CAVSD with associated PDA	6	4.8%
CAVSD with associated DORV	2	1.6%
CAVSD with associated PS	1	0.8%
ASD	6	4.8%
ASD with associated PDA	9	7.3%
TOF	7	5.6%
TOF with associated CAVSD	3	2.4%
TOF with associated DORV	3	2.4%
TOF with associated PDA	2	1.6%
TOF with associated ASD	2	1.6%
PA with associated PDA	2	1.6%

**Table 3:** Mother age at childbirth

Mother age	Number	Percentage
20 years to 25 Years	28	22.7%
26 years to 30 years	33	26.8%
31 years to 35 years	22	17.8%
36 years to 40 years	33	26.8%
Above 40 years	7	5.6%

**Table 4:** Growth failure Ventricular septal defect (VSD), Patent ductus arteriosus (PDA), Atrial septal defect (ASD), Complete atrioventricular septal defect (CAVSD)

Type of CHD	Number	Percentage (among growth failure)
VSD	16	22.8%
VSD with associated PDA	9	12.8%
VSD with associated ASD and PDA	7	10%
VSD with associated ASD	1	1.4%
PDA	14	20%
CAVSD	10	14.2%
CAVSD with associated PDA	6	8.5%
ASD	2	2.8%
ASD with associated PDA	5	7.1%

## Discussion

CHD in DS is reported at around 40 to 63% and is among the leading causes of mortality and morbidity in these children below two years of age [10,12]. It is suggested by studies that the most common lesion observed in children with DS having CHD is variable according to different geographic regions in the world [9,10]. Therefore, it is important to know the profile and association of CHD in DS for a given geographic area for the planning of management like intervention, surgical management and medical follow-up keeping in view the high risk of pulmonary vascular disease in these patients [11,13].

In this study, our aim was to assess the frequency of different types of CHD in DS and its association in Pakistan. As our institute is a center for cardiac patient referral, receiving almost a quarter of the country's population, the results may reflect the national trend of CHD in children with DS.

It was observed in our study that 52.8% patients were male and 47.2% of the patients were female. Most of the patients were diagnosed under one year of age however 10.5% patients had a delayed diagnosis which could be due, in part, to lack of awareness, poverty and lack of access to health services providing facilities. The median age of mothers at the time of birth was 31 years (20-45 Years). Advanced maternal age is a well-known risk factor for acquiring DS which increases to one in 30-50 live birth with mothers aged 45 years and above, although the exact etiology is yet to be defined [14]. However, studies in the general population have suggested no significant increase in the risk of CHD in newborns born to mothers with advanced maternal age [15].

Heart defects vary according to the geographic location of children with DS. In our study the most common isolated CHD was VSD seen in 22.8% patients, followed by PDA in 14.6%, CAVSD in 14.2%, TOF in 5.6%, and ASD in 4.8% patients. The most common associations of CHD were VSD+PDA in 9% patients and ASD+PDA in 7.3% patients. Multiple CHDs were observed in 45.5% of patients. VSD (40%) is also reported to be the most common isolated defect in DS in China, whereas Latin American studies have shown Secundum ASD as the most common lesion [10,16,17]. Studies from European countries and USA have reported AVSD (43%) as the most common defect followed by VSD (32%), secundum ASD (10%), TOF (6%) and PDA (4%). About 30% of patients had several CHDs [3,12]. The result of our study exhibits the same dominant CHD compared to the neighboring country.

About 57% of patients were diagnosed below one year of age, and severe pulmonary hypertension was seen in 45.5% patients. it is important to emphasize that early diagnosis of CHD in DS is critical for reducing morbidity and mortality, keeping in view the early development of pulmonary hypertension [18].

Growth failure was seen in 57% of patients. All patients with growth failure had underlying left to right shunt lesions. El-Shazali et al reported that 56.9% of patients with growth failure [19]. Another study reported that 71% of patients with DS had growth failure compared to 21% in patients with no chromosomal abnormality presenting for cardiac surgery [20]. These results suggest that CHD in DS has a significant effect on the growth of the patients.

This study has limitations because the use of noninvasive assessment tools and cardiac catheter studies would have helped accurately determine pulmonary pressures. It is a single-center study, and more data need to be obtained to further establish the pattern of CHD with DS in the region.

## Conclusions

DS is commonly associated with CHD. Early assessment of patients with DS for CHD is recommended. VSD is the most common type of CHD with DS. The most common association of CHD in DS is VSD, and PDA followed by ASD and PDA. Most of the patients with CHD and DS have growth failure.
